# The Antitumor Efficacy of *β*-Elemene by Changing Tumor Inflammatory Environment and Tumor Microenvironment

**DOI:** 10.1155/2020/6892961

**Published:** 2020-02-19

**Authors:** Qiang Xie, Fengzhou Li, Lei Fang, Wenzhi Liu, Chundong Gu

**Affiliations:** ^1^Department of Thoracic Surgery, The First Affiliated Hospital of Dalian Medical University, Dalian, Liaoning 116011, China; ^2^Lung Cancer Diagnosis and Treatment Center of Dalian, The First Affiliated Hospital of Dalian Medical University, Dalian, Liaoning 116011, China

## Abstract

Inflammatory mediators and inflammatory cells in the inflammatory microenvironment promote the transformation of normal cells to cancer cells in the early stage of cancer, promote the growth and development of cancer cells, and induce tumor immune escape. The monomeric active ingredient *β*-elemene is extracted from the traditional Chinese medicine *Curcuma wenyujin* and has been proven to have good anti-inflammatory and antitumor activities in clinical applications for more than 20 years in China. Recent studies have found that this traditional Chinese medicine plays a vital role in macrophage infiltration and M2 polarization, as well as in regulating immune disorders, and it even regulates the transcription factors NF-*κ*B and STAT3 to alter inflammation, tumorigenesis, and development. In addition, *β*-elemene regulates not only different inflammatory factors (such as TNF-*α*, IFN, TGF-*β*, and IL-6/10) but also oxidative stress in vivo and in vitro. The excellent anti-inflammatory and antitumor effects of *β*-elemene and its ability to alter the inflammatory microenvironment of tumors have been gradually elaborated. Although the study of monomeric active ingredients in traditional Chinese medicines is insufficient in terms of quality and quantity, the pharmacological effects of more active ingredients of traditional Chinese medicines will be revealed after *β*-elemene.

## 1. Introduction

*Curcuma wenyujin*, a kind of traditional Chinese medicine that has been planted and used for thousands of years, belongs to Zingiberaceae and has an oval, long oval, or spindle shape [1]. The good cholagogic action and analgesic and bactericidal effects of *Curcuma wenyujin* have been recorded in the *Compendium of Materia Medica* [2, 3]. Current studies have shown that it also has antioxidant, antiproliferation, and antitumor effects [4, 5].

**Figure 1:**
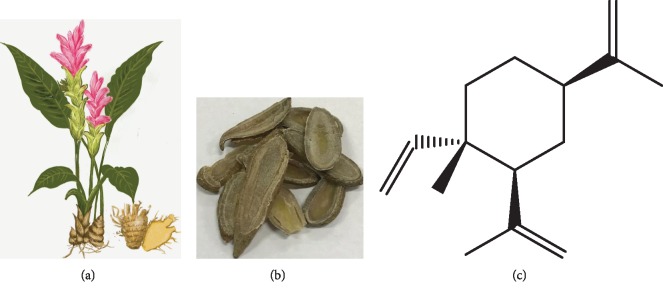
(a) Curcuma wenyujin, a green plant of family Zingiberaceae, is the source of elemene. (b) The traditional Chinese medicine turmeric, taken from the roots of Curcuma wenyujin. (c) The molecular structure of effective monomer components of *β*-elemene.

Elemene, a sesquiterpene compound extracted from *Curcuma wenyujin*, is composed of two essential elements, carbon and hydrogen [6]. The chemical formula of elemene is C_15_H_24_, and the molecular mass is 204.355. *β*-Elemene (1-methyl-1-vinyl-2,4-diisopropenyl-cyclohexane) is the main active ingredient among all three monomer forms of elemene: *α*, *β*, and *δ* [7] (Figure 1). It is a noncytotoxic class II antineoplastic drug that was developed in China with a new structure and has many outstanding advantages, such as broad antineoplastic effects, exact curative effects, low toxicity and side effects, and low resistance [6, 8, 9].

Research has found that *β*-elemene has direct antitumor effects, and its antitumor mechanisms include inducing apoptosis [10], arresting the cell cycle [11], inhibiting angiogenesis and cell migration [12], enhancing the immunogenicity of tumor cells [13], promoting erythrocyte immune function, and inhibiting cancer stem cell-like effects [14]. *β*-Elemene not only has direct antitumor effects but also reverses multidrug resistance by reducing mitochondrial membrane potential, activating the intracellular redox system, and inducing apoptosis of tumor cells [10, 15]. *β*-Elemene increases chemosensitivity by inducing tumor cell apoptosis and increases the sensitivity to radiotherapy by inhibiting the P21-activated kinase1 (PAK1) signaling pathway [16, 17], inducing DNA damage, and restraining DNA damage repair [18].

Currently, *β*-elemene and its derivatives have been utilized to treat various tumors including lung cancer [10], liver cancer [11], brain cancer [19], breast cancer [20], ovarian cancer [21], stomach cancer [16], prostate cancer, and other tissues for over 20 years [22]. Their practical and effective medicinal value has been confirmed gradually.

A total of 120 patients with refractory/relapsed acute myeloid leukemia (AML) were treated with HAA (homoharringtonine, arabinosylcytosine, and aclacinomycin) combined with *β*-elemene. The total effective rate of the *β*-elemene emulsion plus HAA group was 80.8%, which was significantly better than that of the control group (52.9%). This result indicated that *β*-elemene had a synergistic effect on acute myelogenous leukemia [23].

In 2019, a meta-analysis of 15 randomized controlled trials (RCTs) in accordance with PRISMA guidelines recruited 1410 patients with stage III/IV non-small-cell lung cancer (NSCLC) and found that elemene improved clinical efficacy, enhanced cellular immune function, and reduced the toxicity of chemotherapy. It was confirmed that *β*-elemene was a safe and effective adjuvant therapy for platinum-based chemotherapy in stage III/IV NSCLC patients [24]. In 2018, Wang et al. pooled 46 controlled clinical trials with a total of 2992 patients. The results showed that *β*-elemene significantly increased the overall efficacy of controlling malignant pleural effusion without increasing the incidence of chest pain and fever [25].

In addition, Xu et al. [9], Wang et al. [26], Jiang et al. [27], and Xu et al. [28] also confirmed the efficacy and safety of *β*-elemene in clinical use over the past 20 years.

## 2. The Inflammatory Microenvironment and Tumorigenesis

### 2.1. Overview of Inflammation and Tumors

There is an abnormal relationship between the tumor parenchyma and its surrounding microenvironment [29]. The interstitial cells of the tumor are composed of two major types: cellular components and noncellular components [30]. The interstitium of the tumor participates in the interaction of the tumor parenchyma to determine the biological behavior of the tumor [31]. When a pathogen enters the host, it causes damage to the organism and activates the immune system [32, 33]. Moreover, a large number of inflammatory cells, such as tumor-related macrophages and dendritic cells, infiltrate and activate [34]. These cells also promote each other with tumor-related inflammatory cells, which results in a variety of tumorigenic factors in the tumor microenvironment [35]. These changes promote the growth of tumor parenchyma and the formation of the tumor interstitial blood vessels and destroy the immune system of the body, resulting in the transformation of the interstitium and the metastasis of tumors [31, 36, 37].

### 2.2. Changes in the Tumor Inflammatory Environment

When the body is infected or repairs wounds, it permanently activates and chemotactically accumulates a large number of white blood cells (such as macrophages, neutrophils, lymphocytes, and dendritic cells) at the site of injury by releasing cytokines/chemokines (such as interleukin-6/10 (IL-6/10) [38, 39] and tumor necrosis factor-*α* (TNF-*α*)) [40], growth factors (transforming growth factor-*β* (TGF-*β*)) [41], matrix metalloproteinases (MMPs) [42], vascular endothelial growth factor (VEGF) [43], reactive oxygen species (ROS) metabolites, and other substances [44]. These inflammatory cytokines not only recruit inflammatory cells to amplify inflammation at the tumor site [45] but also form a new environment [46], leading to the destruction and atrophy of normal tissues [47] and promoting mass production of the tumor matrix and blood vessels [48]. These factors play an essential role in the occurrence and development of tumors and promote the growth and metastasis of tumors. Inflammation leads to cell transformation, and the tumor cells and their surrounding interstitium secrete cytokines and chemical activators, forming a positive cycle between cancer and inflammation, which is conducive to the communication between tumors and the host stroma, thus accelerating the progression of tumors [49, 50].

TNF-*α* is a special and multifunctional cytokine that plays a key role in immune regulation, the inflammatory response, and defense [51]. On the one hand, a high concentration of TNF-*α* destroys tumor blood vessels, causes cell necrosis, and also stimulates tumor-specific T cells, which have an antitumor effect. In vitro studies have shown that TNF-*α* directly kills various human tumor cells, such as melanoma, breast cancer, and cervical cancer cells [52]. On the other hand, the role of TNF-*α* in chronic inflammation and its tumor-promoting effect have also been proven [53]. In human tumors such as bladder cancer, prostate cancer, colon cancer, leukemia, and lymphoma, elevated TNF-*α* levels have been detected [54]. In the tumor microenvironment, TNF-*α* is secreted by macrophages and tumor cells. Continuous TNF-*α* stimulation promotes tumor angiogenesis, DNA damage, tumor epithelial-mesenchymal transition (EMT), and other mechanisms to promote tumor survival and metastasis [55], and its mechanism may be related to the activation of the nuclear factor kappa-B (NF-*κ*B) signaling pathway [56]. TNF-*α* treatment of tumor cells that were then intravenously injected into nude mice significantly enhanced their tumorigenicity [57]; at 9 days after tail vein injection of cancer cells in nude mice, LPS stimulation not only significantly improved the level of TNF-*α* but also increased the number of lung metastases [58]. These studies show that TNF-*α* plays an important role in the inflammatory environment and tumor microenvironment. Moreover, we hypothesize that the tumor-promoting or anticancer response of TNF-*α* in the tumor microenvironment depends not only on the local concentration but also on its expression source in the tumor.

As an inflammatory cytokine, IL-6 is mainly derived from stromal cells such as macrophages and fibroblasts around the tumor, and it is not secreted or is rarely secreted by the tumor cells themselves [59, 60]. IL-6, similar to TNF-*α*, promotes the transformation of noncancer cells into cancer stem cells [61] while regulating the biological activity of tumor cells, leading to cell proliferation or distant metastasis [62]. In addition, IL-6 promotes tumor development by enhancing the T cell-mediated immune-inflammatory response, regulating gene expression, and inhibiting apoptosis in the cell through the JAK-STAT signaling pathway [63, 64].

### 2.3. Macrophages and Tumors

There are many inflammatory cells involved in the initiation, development, and metastasis of cancer, of which tumor-associated macrophages (TAMs) account for the largest proportion (50% of inflammatory cells) and play the most significant role [65, 66]. Macrophages secrete a variety of cytokines and cytotoxic mediators, including activator of transcription 3 (STAT3), colony-stimulating factor receptors (CSF-1), ROS, and MMP [67], which promote not only abnormal proliferation and apoptosis of early cells but also the formation and development of tumors and accelerate the infiltration and metastasis of tumor cells [68]. Studies have shown that M1 macrophages are the main type in the early stage of inflammation, and the M2-type macrophages are mainly found in the late stage [69]. In addition, M1 macrophages can transform into M2 macrophages, which have immunosuppressive functions to accelerate the malignant transformation of benign tumors [70]. Therefore, the previous understanding of the functions of M1 and M2 macrophages is not altogether true, but we can be sure that macrophages are crucial in the transformation of the tumor microenvironment and tumorigenesis.

### 2.4. Immune Function and Tumors

The immune characteristics of the tumor microenvironment have been listed as one of the ten characteristics of the tumor [71]. Studies have shown that the tumor is locally infiltrated with a variety of immune cell subsets. Macrophages, mast cells, dendritic cells (DCs), and myeloid-derived suppressor cells (MDSCs) are distributed in the central area and around the tumor. NK cells are mainly located in the tumor matrix. Immature DCs are mainly located in the central area of the tumor, while mature DCs and B cells are mostly found in secondary lymphoid tissue. In addition, CD8^+^ T cells are mainly distributed at the edge of the tumor [72, 73].

The body's normal immune system is able to recognize and remove foreign cells or cancerous cells [74]. However, in the inflammatory microenvironment, dynamic changes in inflammation and immune abnormalities develop, which mainly manifest as inflammation-induced immunosuppression and immune escape [75]. Tumor cells also secrete a variety of immunosuppressive factors. The inhibitory effects of TGF-*β*1 and IL-10 are relatively strong [76, 77]. Immunosuppressive factors activate inflammatory mediators, such as macrophages, mast cells, and natural killer (NK) cells, to secrete IL-10, IL-6, and TNF-*α* [78]. The vicious cycle is formed in the inflammatory microenvironment, which induces tumor immunosuppression to some extent.

### 2.5. Oxidative Stress and Tumors

Infection or chronic inflammation contributes to the occurrence of cancer mainly by leukocytes and immune cells in inflammatory lesions, which are activated by inflammation and produce ROS and reactive nitrogen species (RNS) [79, 80]. ROS cause damage by oxidizing DNA (including point mutations, deletions, and gene reassortment), disrupting DNA repair, and posttranslationally modifying cancer proteins [81]. DNA damage is increased by the secretion of MIF from macrophages and T lymphocytes [82]. When cells are exposed to persistent oxidative stress caused by chronic inflammation, the RNS nitric oxide (NO) induces gene mutation and inactivates key enzymes for DNA damage repair, thereby preventing or impairing DNA repair and exacerbating DNA damage [83, 84]. NO is an important inflammatory mediator that is associated with chronic inflammation and cancer and is produced endogenously through different isomerizations of nitric oxide synthase (NOS) during arginine metabolism [85, 86]. During inflammation, macrophages and epithelial cells induce iNOS expression. In the inflammatory microenvironment, local iNOS activity is induced for a short time, resulting in increased NO of more than 10^3^ times the basal level [87]. The local increase in NO is easily oxidized by ROS to produce nitrogen peroxide (NOO), which is a RNS. In the clinic, many precancerous lesions and cancers have elevated levels of iNOS and NO [85]. The endogenous NO produced by tumor cells and tumor vascular endothelial cells plays an important role in promoting tumor angiogenesis and ensuring the maximum blood supply of tumors. Tumor angiogenesis is the basis of tumor growth and metastasis [88].

In addition, there is a dual relationship between NO and tumors: an appropriate concentration of NO promotes tumor growth, while a high concentration of NO is not conducive to tumor growth and has an antitumor effect [89]. In general, the concentration of NO that has an antitumor effect is 10-100 times higher than the concentration that promotes tumor growth [90]. On the one hand, NO mediates the tumor-killing effect of macrophages. NO is more effective than TNF-*α* in mediating the killing of tumor cells by activated macrophages [91]. On the other hand, NO directly kills tumor cells: (1) NO acts on mitochondrial oxidoreductase, and tumor cells die due to energy metabolism disorders [92]; (2) NO combines with superoxide anion in cells to generate nitrogen/oxygen free radicals, resulting in genotoxicity and DNA damage [93]; (3) NO inhibits cell proliferation by inhibiting protein synthesis [94]; (4) NO activates the expression of p53 and induces apoptosis of tumor cells [94]; and (5) NO inhibits platelet aggregation and tumor metastasis [95]. In addition, a large number of studies have found that NO is also widely involved in the chemotherapy and immunotherapy of tumors, interacting with chemotherapy drugs and cytokines and affecting the efficacy of drugs against tumors [96].

### 2.6. Tumor-Promoting Pathways

The transcription factors NF-*κ*B and STAT3 are involved in inflammation and tumorigenesis and regulate cell survival, growth, and proliferation [97]. Many cytokines involved in the inflammatory response, such as TNF and IL-1, are involved in the activation of the NF-*κ*B signaling pathway [88]. Activation of the STAT3 pathway also primarily depends on the corresponding inflammatory cytokines, such as IL-6, IL-10, and VEGF [98, 99].

**Figure 2:**
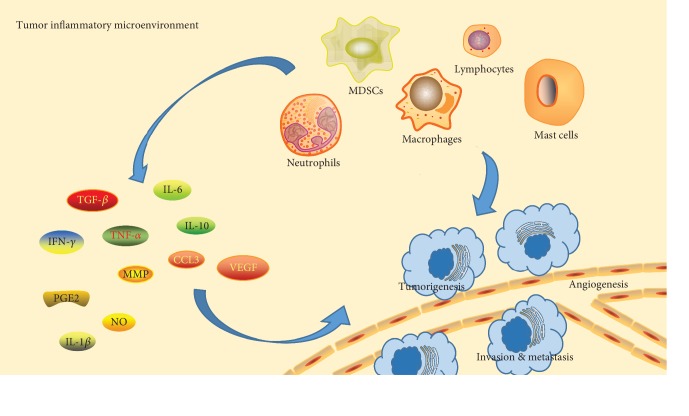
Effect of inflammatory factors and inflammatory cells on tumor development in the inflammatory microenvironment.

There are some crossovers and interactions between these two signaling pathways. After activating these two signaling pathways in inflammatory cells, cytokines, chemokines, and enzymes related to the synthesis of prostaglandins and inducible nitric oxide synthase are released to form an inflammatory microenvironment that is conducive to tumorigenesis [100, 101]. In malignant transformed cells, these two signaling pathways promote malignant proliferation, enhance adhesion and promote the expression of antiapoptotic genes such as Bcl-2 [102], and play a key role in the production, survival, epithelial-mesenchymal transition (EMT), invasion, and metastasis of cancer cells by inhibiting adaptive immunity and drug resistance [103]. These two signaling pathways play a crucial role in bridging tumor cells and peripheral inflammatory cells (Figure 2).

## 3. *β*-Elemene Alters Inflammation and the Tumor Microenvironment

### 3.1. Regulation of Inflammatory Factors by *β*-Elemene

TNF is a cytokine that directly kills tumor cells and has no obvious toxicity to normal cells, and it is one of the most potent biologically active factors [52]. In many injury models, elevated expression of TNF-*α* is associated with tissue damage [54]. *β*-Elemene decreases the levels of endotoxin in plasma, TNF-*α* in serum, and CD14 in the liver of rats with liver fibrosis, preventing concurrent liver fibrosis induced by carbon tetrachloride (CCl_4_) and reducing liver injury and inflammatory reactions [104, 105]. Elemene reduces not only macrophage infiltration in inflammation but also the production of TNF-*α* and IL-6 by macrophages to alleviate endothelial damage and delay atherosclerosis [106]. In lipopolysaccharide- (LPS-) stimulated RAW264.7 macrophages, *β*-elemene inhibited *β*-catenin in a dose-dependent manner and inhibited the upregulation of IL-6, TNF-*α*, and IL-1*β* under LPS stimulation, thereby confirming the importance of the Wnt/*β*-catenin signaling pathway in the anti-inflammatory activity of *β*-elemene [106]. The decrease in TNF-*α*, IL-1*β*, IL-6, IL-8, and other inflammatory factors was detected after treatment of LPS-stimulated macrophages and neutrophils with elemene, which shows that the anti-inflammatory activity of *β*-elemene is similar to that of dexamethasone and indicates that elemene has a strong inhibitory effect on the inflammatory response [107–109]. When evaluating the immunoregulatory activity of neutrophils stimulated by LPS in vitro, it was found that reducing the generation of MMP-9 and TNF-*α* protected tissues from the proteolytic activity of matrix-degrading enzymes released by neutrophils and inhibited neutrophil migration [108]. The reduction in MMP-9 affected the release of TGF-*β*1, neutrophil chemotaxis, and VEGF [110].

The biological functions of TGF-*β* were initially studied in inflammation, tissue repair, and embryonic development [111]. Recently, it has been found that TGF-*β* plays an important role in regulating cell growth, differentiation, and immune function [112, 113]. In contrast to the effects on normal human airway fibroblasts, *β*-elemene dose-dependently inhibits the release and expression of Wnt3a, inactive GSK-3*β*, *β*-catenin, *α*-SMA, TGF-*β*, and Col-1 in human airway granulation tissue fibroblasts and has the same effect on the expression and nuclear translocation of active *β*-catenin [114]. Therefore, the effect of *β*-elemene on primary human airway granulation tissue fibroblasts may occur through the downregulation of the classical Wnt/*β*-catenin and TGF-*β*/Smad pathways [115, 116]. These pathways may be promising targets for improving benign airway stenosis and inhibiting excessive proliferation of fibroblasts. TGF-*β* and Col-1 are two important molecules that are secreted by fibroblasts and also induce fibroblasts to promote inflammation and cancer. The TGF-*β*-induced upregulation of *α*-SMA and CD44 in LX-2 cells was blocked by *β*-elemene [117]. In addition to CD44, *α*-SMA is produced when fibroblasts are stimulated and is essential for cell movement, tumor development, and invasion [118, 119].

### 3.2. *β*-Elemene Protects Immune Disorders

Progressive growth and immune escape in most malignant tumors occur when tumor antigens cannot be effectively presented to T cells to induce antigen-specific immune response [120, 121]. When *β*-elemene is combined with immunotherapy, a large number of inflammatory cells infiltrate tumor tissue and enhance dendritic cell (DC) antigen presentation, which may be one of the mechanisms by which *β*-elemene exerts antitumor immunity and counteracts tumor immune escape [122, 123]. When bone marrow-derived dendritic cells (BM-DCs) modified with the murine IL-23 gene were used in combination with elemene in pancreatic cancer model mice, we found that the combination therapy significantly increased the inhibition of tumors and enhances the specific Th1 and cytotoxic T lymphocyte (CTL) responses [124, 125]. This combination treatment significantly promoted the secretion of interferon-*γ* (IFN-*γ*) and had antiviral, antitumor, and immunoregulatory effects, and IFN-c inhibited the expression of IL-4 in vitro and in vivo [122, 124]. This effect against immune escape and the antitumor synergy of *β*-elemene are the focus of our future studies.

**Figure 3:**
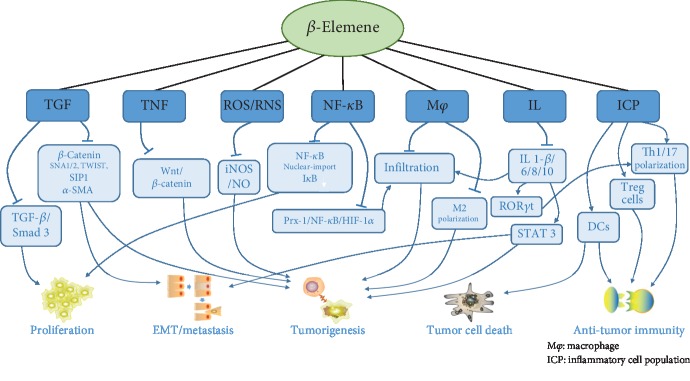
Overview of the mechanism by which *β*-elemene changes the inflammatory environment to regulate inflammatory processes and tumor development.

Immune disorders in tumors are often seen in demyelinating diseases. *β*-Elemene regulates the immune balance through the blood-brain barrier [126], inhibits the downregulation of Treg cells and Th17 and Th1 polarization, and downregulates the expression of the proinflammatory factor IL-17, which has a substantial protective effect on optic nerve inflammation in experimental autoimmune encephalomyelitis [127]. In an experimental autoimmune encephalomyelitis mouse model, we observed that *β*-elemene selectively downregulated CD4+ T lymphocytes without affecting the activation of peripheral lymphoid tissue, significantly weakened the signs in the nervous system and the development of experimental autoimmune encephalomyelitis (EAE), inhibited the Th1 cell-mediated immune response, and upregulated the Treg cell response in vitro [128]. Improvement of EAE by *β*-elemene may depend on inhibition of IL-6-activated ROR*γ*t signal transduction, the STAT3 pathway, and promotion of Treg cell proliferation to inhibit the development and differentiation of Th17 cells [129]. Therefore, *β*-elemene control of inflammatory diseases mediated by Th17 cells and other cells and regulation of tumor immunity disorder are of great significance (Figure 3).

### 3.3. *β*-Elemene Regulates NF-*κ*B/STAT3

There is a strong link between the long-term inflammatory response and cancer, and an important mediator of the inflammatory response and cancer is NF-*κ*B. Inhibition of the NF-*κ*B signaling pathway may be one of the important mechanisms by which *β*-elemene changes the inflammatory environment and tumor microenvironment. In LPS-stimulated macrophages, expression of NF-*κ*B (p65) decreased in the *β*-elemene treatment group [106], which may be related to the elemene-mediated inhibition of the expression of Toll-like receptor 4 (TLR4), iNOS, cyclooxygenase-2 (COX-2), TNF-*α*, IL-1*β*, IL-6, and IL-12 [107]. *β*-Elemene not only reduces the expression of NF-*κ*B but also inhibits its transport to the nucleus; together with inhibition of the RAC1/MLK3/p38 signaling pathway, this may be one of the mechanisms by which elemene alleviates septicemia-related encephalopathy (SAE) [109]. As a radiosensitizer of lung cancer, *β*-elemene effectively controls radiation- and hypoxia-induced activation of the Prx-1/NF-*κ*B/HIF-1*α* pathway, inhibiting the expression of monocyte chemoattractant protein 1 (MCP-1) and the infiltration and polarization of M2 macrophages induced by radiation in vivo, which reduces the damage and improves the inflammatory environment of tumors [130]. Since *β*-elemene easily passes through the blood-brain barrier, the expression of inflammatory factors, TLR4 and Caspase-3, is significantly decreased, and the expression of I*κ*B is upregulated when traumatic brain injury (TBI) is treated with *β*-elemene alone or in combination with hyperbaric oxygen (HO) [131], which exhibits an anti-inflammatory effect and neuroprotection by inhibiting the NF-*κ*B signaling pathway. In the Th1 cell-mediated EAE animal model, *β*-elemene improves the course of EAE mice by inhibiting ROR*γ*t, which is activated by the IL-6 and STAT3 pathways [129]. The NF-*κ*B and STAT3 pathways play key roles in the formation of the inflammatory environment, as well as in the development, invasion, and metastasis of tumors [97]. The inhibitory effect of *β*-elemene on these two pathways has shown good anti-inflammatory and anticancer applications, but this requires further research.

### 3.4. Effects of *β*-Elemene on Macrophages

Macrophages are the core participants in inflammation and the immune response and are involved in a variety of disease processes [65]. One of the most effective stimuli for macrophages is the bacterial endotoxin LPS [132]. When macrophages are exposed to LPS, TLR4 recognizes LPS and induces the production of many inflammatory cytokines, such as TNF-*α*, IL-6, and IL-1*β*. Treatment with *β*-elemene reduces the expression of TLR4, suggesting that elemene has anti-inflammatory activity in LPS-stimulated macrophages [107]. *β*-Elemene inhibits the LPS-induced expression of iNOS and IL-10 by inhibiting *β*-catenin and downregulating the Wnt/*β*-catenin signaling pathway [106]. *β*-Elemene effectively inhibits the synthesis of COX-2 and prostaglandin E2 (PGE2), and the concentrations of LPS-induced IL-1*β*, IL-6, TNF-*α*, and IL-12 also decrease with the downregulation of iNOS and NO [107], demonstrating the crucial role of *β*-elemene in the congenital and inflammatory responses of macrophages triggered by TLRs. In atherosclerotic lesions, macrophage foam cells contribute to the formation of fatty streaks, which is an essential event in the eventual formation of atherosclerotic plaques [133]. *β*-Elemene reduces macrophage infiltrations, inhibits the production of TNF-*α* and IL-6 in macrophages, and reduces serum total cholesterol (TC), triglycerides (TGs), and low-density lipoprotein (LDL-C) in vivo [105], regulating the level of blood lipids. Moreover, ROS are mainly produced by macrophages, and the decrease in macrophage infiltration also reduces endothelial oxidative stress injury and delays the progression of atherosclerosis [134].

Macrophages can be divided into two different phenotypes: the M1 phenotype, which is the classically activated phenotype that is involved in antitumor immunity, and the M2 phenotype, which is an alternatively activated phenotype with tumor-promoting properties [135]. A number of studies have found that TAMs are mostly M2-polarized, and it has been reported that these cells promote the growth and survival of tumors and may lead to resistance to cancer treatment [65]. *β*-Elemene not only decreases the proliferation, migration, and invasion and strengthens the radiosensitivity of lung cancer cells but also inhibits the promotion of migration, invasion, and EMT of lung cancer in M2 macrophage-conditioned medium, regulating the polarization of macrophages from M2 to M1 [136]. Research shows that tumor cells in the irradiated or hypoxic microenvironment recruit macrophages and induce MCP-1 secretion, which leads to nuclear accumulation of NF-*κ*B and HIF-1*α*. *β*-Elemene significantly controls the infiltration and polarization of M2 macrophages and MCP-1 secretion induced by radiation and hypoxia by inhibiting the Prx-1/NF-*κ*B/HIF-1*α* pathway [130]. MCP-1 is an important proinflammatory cytokine that is secreted by macrophages, monocytes, and fibroblasts during inflammation, and it has specific chemotactic effects on monocytes/macrophages [137]. *β*-Elemene inhibits macrophage infiltration in patients undergoing radiotherapy and increases the radiosensitivity of lung cancer cells by enhancing DNA damage, inhibiting DNA repair, or causing apoptosis of radiation cells, which is a promising strategy for chemotherapy or radiotherapy [10, 18]. *β*-Elemene participates in the defense against Leishmania through increased phagocytosis and lysosomal activity, which may be related to the activation of M1 macrophages by *β*-elemene [138]. *β*-Elemene plays important anti-inflammatory and antitumor roles in inhibiting the activation and invasion of macrophages, and the effects of the inflammatory environment and tumor microenvironment on macrophages deserve further study.

### 3.5. *β*-Elemene Changes iNOS and NO Levels

iNOS, inducible nitric oxide synthase, is a catalytic enzyme that is produced by NOS active nitrogen free radicals in the body [86], and the level of iNOS may be an important indicator of the degree of inflammation in an organism [87]. In the inflammatory microenvironment, macrophages and epithelial cells induce the expression of iNOS, and then, the local iNOS activity in the inflammatory microenvironment is significantly increased quickly, leading to an increase in NO of more than 10^3^ times the basal state [139]. NO participates in the regulation of physiological and pathophysiological processes such as vascular and nervous system functions and has cytotoxic effects at high concentrations [85]. Elemene treatment of NCI-H292 cells, a human lung adenocarcinoma cell line, increased the levels of p38 mitogen-activated protein kinase (MAPK) and iNOS, and so, a locally increased concentration of NO may be associated with elemene-induced apoptosis [140]. In a mouse model of experimental autoimmune encephalomyelitis, elemene restrained microglial activation and iNOS expression, which was associated with the inhibition of axonal demyelination and neuronal death during the development of the disease [128]. *β*-Elemene effectively downregulates iNOS expression and inhibits NO production; furthermore, inhibition of iNOS leads to a decrease in PGE2 synthesis and downregulation of COX-2 expression, which indicates that elemene has strong anti-inflammatory activity in LPS-stimulated macrophages [107, 141]. The inhibition of iNOS and IL-10 by *β*-elemene inhibits *β*-catenin activity in a dose-dependent manner and downregulates the Wnt/*β*-catenin signaling pathway [106]. *β*-Elemene increases the activity of T-AOC, SOD, CAT, and GSH-Px, suggesting that *β*-elemene enhances the removal of free radicals and protects cells from oxidative damage caused by free radicals [142]. By inhibiting the activation of the MAPK signaling pathway, *β*-elemene significantly inhibits the production of ROS and inhibits hydrogen peroxide-induced apoptosis of human umbilical vein endothelial cells (HUVECs), enhancing the viability of damaged cells [143, 144]. The potential value of *β*-elemene in effectively resisting oxidative stress associated with cardiovascular diseases deserves further study.

NO expression in endothelial cells exerts antiproliferative, anti-inflammatory, and antioxidative effects in the vascular wall. *β*-Elemene increases the production of nitric oxide (NO) in HUVECs by significantly improving plasma nitrite and nitrate levels and promotes the phosphorylation of eNOS (ser1177) and Akt in vitro to maintain endothelial function [145]. These data show that *β*-elemene acts through its antioxidant and anti-inflammatory characteristics to affect atherosclerosis and enhanced plaque stability. In mouse models of arterial injury, *β*-elemene effectively controls the proliferation and migration of VSMCs and inhibits neointimal formation in vivo [146]. These factors are associated with oxidative stress and VSMC dilation and reveal the potential clinical application of *β*-elemene in preventing vascular stenosis and remodeling. There is no obvious cytotoxicity of *β*-elemene or a series of its derivatives. These factors have the ability to increase superoxide dismutase activity and nitric oxide secretion in cells and simultaneously decrease malondialdehyde content and lactate dehydrogenase release in cells; these changes mediate antioxidant activity [147]. In addition, the regulation of biochemical substances (SOD, MDA, NO, and LDH) in HUVECs treated with hydrogen peroxide is better than that of the positive control vitamin [148], and so, *β*-elemene may be a potential treatment to effectively resist oxidative stress associated with cardiovascular disease. *β*-Elemene and some derivatives produce high levels of NO in vitro, and its antitumor activity in U87 cells was significantly attenuated by NO scavengers (hemoglobin or carboxyl-PTIO), and blocking activation of the PI3K/Akt pathway induced G2 arrest of the cell cycle and apoptosis in U87 cells, inhibiting tumor growth [149, 150].

### 3.6. The Relationship between *β*-Elemene and EMT

TGF-*β* is a potent inflammatory factor, as well as a strong activator of the EMT, which is involved in the progression and metastasis of cancer [151]. *β*-Elemene reduces the expression and phosphorylation of Smad3 to inhibit the expression of nuclear transcription factors (such as SNAI1, SNAI2, TWIST, and SIPI1) and block the EMT induced by TGF-*β*1 in the human breast cancer cell line MCF-7 [116]. TGF-*β*1 induces the upregulation of *α*-SMA in human hepatic stellate cells, and its expression and EMT phenotypic transformation can be blocked by *β*-elemene [117]. TLR4 induces the EMT and the production of antiapoptotic proteins and angiogenesis factors, promoting the survival of cancer cells and inducing immunosuppression [152]. The expression of TLR4 in the TBI rat model is significantly downregulated by *β*-elemene and changes the expression levels of Caspase-3 and I*κ*B [131]. The combination of elemene and gefitinib profoundly impairs epithelial cell transformation to mesenchymal cells, in a large part due to the regulation of the enhancer of zeste homolog 2 (EZH2), a carcinogenic histone methyltransferase and gene transcription regulator, thereby modulating the subsequent effector molecule required for cancer progression [153]. *β*-Catenin may also be the target of *β*-elemene in reversing the malignant phenotype of tumor cells; moreover, notch1, sonic hedgehog, and the epithelial marker of E-cadherin are upregulated by *β*-elemene in human glioblastoma cells in vitro and in vivo [19]. *β*-Elemene-mediated blockade of the phenotype transition of type 3 EMT in metastatic malignant tumors under the continuous stimulation of inflammation may also be an important antitumor mechanism of *β*-elemene.

## 4. Conclusion

It is well known that inflammatory factors in the inflammatory microenvironment are closely related to the development of inflammatory cells and tumors, and the regulation of the inflammatory microenvironment is also involved in the regulation of inflammation and tumor development. *β*-Elemene, an effective monomer extracted from the traditional Chinese medicine *Curcuma wenyujin*, has been applied clinically for more than 20 years in China and exhibits good antitumor and anti-inflammatory activities without obvious cytotoxicity or clinical side effects. The ability of *β*-elemene to regulate the inflammatory environment of tumors has also been demonstrated in recent research. *β*-Elemene regulates many important inflammatory factors (such as TNF-*α*, IFN, TGF-*β*, and IL-6/10), similar to dexamethasone, indicating that it has robust anti-inflammatory and regulatory abilities in the inflammatory environment of tumors. *β*-Elemene downregulates the levels of iNOS and NO, regulates oxidative stress in vivo and in vitro, alleviates tissue damage, and inhibits the formation of the microenvironment that promotes tumorigenesis. Under certain conditions, elemene also increases the ability of iNOS, which results in apoptosis induced by a locally increased NO concentration, but its specific mechanism needs to be further explored. The NF-*κ*B and STAT3 pathways play key roles in the formation of the inflammatory environment and the occurrence, development, invasion, and metastasis of tumors. *β*-Elemene not only reduces the expression of NF-*κ*B and inhibits its translocation to the nucleus but also upregulates the expression of I*κ*B. However, beyond that, *β*-elemene influences the inflammatory microenvironment of tumors and inflammation and tumor progression by inhibiting the IL-6-induced ROR*γ*t and STAT3 pathways. The inhibitory effect of *β*-elemene on these two pathways shows good anti-inflammatory and anticancer application prospects. Immune escape is an important reason for the rapid growth and metastasis of tumors. Elemene plays an important role in inhibiting macrophage infiltration and M2 polarization, as well as in regulating immune disorders. The modern pharmacological mechanism of elemene as an antineoplastic drug and radiosensitizer is gradually becoming understood. We hope that more effective monomers of traditional Chinese medicine will gain attention and that more traditional Chinese medicines with thousands of years of application history will be verified by modern pharmacology.
